# 4D flow MRI-based grading of left ventricular diastolic dysfunction: a validation study against echocardiography

**DOI:** 10.1007/s00330-025-11703-0

**Published:** 2025-05-25

**Authors:** Clemens Reiter, Gert Reiter, Ewald Kolesnik, Daniel Scherr, Albrecht Schmidt, Michael Fuchsjäger, Ursula Reiter

**Affiliations:** 1 Division of General Radiology, Department of Radiology, Medical University of Graz, Graz, Austria; 2 Division of Neuroradiology, Vascular and Interventional Radiology, Department of Radiology, Medical University of Graz, Graz, Austria; 3 Division of Cardiology, Department of Internal Medicine, Medical University of Graz, Graz, Austria; 4 Research and Development, Siemens Healthcare Diagnostics GmbH, Graz, Austria

**Keywords:** Magnetic resonance imaging, Diagnostic imaging, Validation study, Left ventricular dysfunction, Diastole

## Abstract

**Objectives:**

To assess the feasibility and accuracy of 4D flow MRI-based grading of left ventricular diastolic dysfunction, using echocardiography as the reference method.

**Methods:**

Between October 2016 and February 2022, subjects were prospectively recruited for transthoracic echocardiographic evaluation of left ventricular diastolic function and 4D flow MRI at 3 T. Echocardiographic grading of diastolic dysfunction was performed according to the multiparametric, threshold-based 2016 ASE/EACVI approach. Volumetric and echo-equivalent peak velocity grading parameters were evaluated from 4D flow magnitude and velocity data, respectively. Duration of vortical blood flow along the main pulmonary artery (*t*_vortex_) was employed as a surrogate grading parameter for echocardiographic tricuspid regurgitant peak velocity (TR). Correlations between grading parameters were analysed; agreement in grading of diastolic dysfunction between methods was assessed using a 5 × 5 contingency table analysis.

**Results:**

The study population consisted of 94 participants (mean age, 62 ± 12 years, 50 females, 34 with structural heart disease). All volumetric and echo-equivalent 4D flow grading parameters demonstrated strong to very strong correlations with echocardiography (*r* = 0.75–0.92). Volumetric parameters showed significant biases between 4D flow and echocardiography. Employing bias-adjusted 4D flow grading cutoffs for volumetric parameter, echo-equivalent cutoffs for diastolic transmitral and myocardial peak velocities, and *t*_vortex_ > 15% as a surrogate cutoff for TR > 2.8 m/s, nearly perfect agreement in diastolic dysfunction grading between methods was observed (weighted kappa = 0.84). There was no evidence for over- or underestimation of grades by 4D flow (*p* = 0.53).

**Conclusion:**

Grading of left ventricular diastolic dysfunction from a single 4D flow measurement is feasible and shows nearly perfect agreement with echocardiography.

**Key Points:**

***Question*** *The lack of comparison studies with echocardiography currently limits cardiac MRI-based grading of diastolic dysfunction. Could 4D flow MRI serve as a viable technique*?

***Findings*** *A single 4D flow MRI measurement allows multiparametric grading of left ventricular diastolic dysfunction in nearly perfect agreement with echocardiography*.

***Clinical relevance*** *Agreement between 4D flow MRI and echocardiographic grading of left ventricular diastolic dysfunction is comparable to that observed in repeated echocardiographic evaluations, suggesting 4D flow as a viable alternative to echocardiography in selected patients, especially when comprehensive MRI is already performed*.

**Graphical Abstract:**

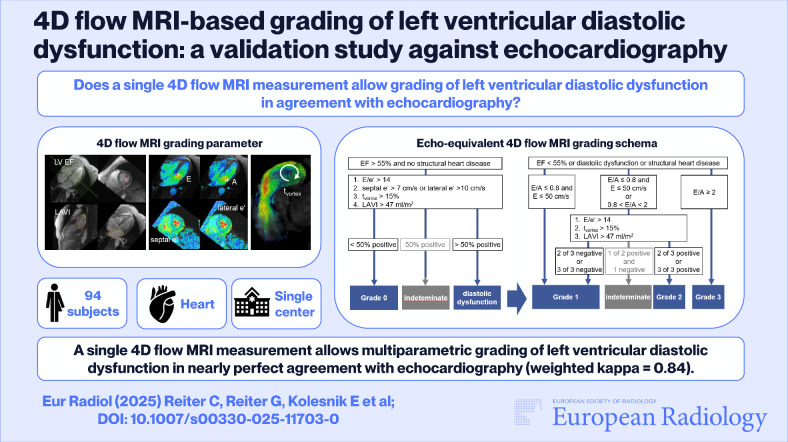

## Introduction

The diagnosis and grading of left ventricular (LV) diastolic dysfunction play an important role in the management and treatment of patients with heart failure. Affecting up to 50% of the population with heart failure, diastolic heart failure is highly prevalent and is associated with significant morbidity and mortality [1].

The American Society of Echocardiography (ASE) and the European Association of Cardiovascular Imaging (EACVI) recommend echocardiography as the method of choice for noninvasive evaluation of LV diastolic (dys)function [2]. The evaluation uses a multiparametric, threshold-based algorithm that includes two-dimensional (2D) and Doppler parameters. Echocardiography, however, has limitations, including poor acoustic window quality, reliance on geometric assumptions, non-detection of tricuspid regurgitation, and dependence on the operator’s expertise, potentially limiting the assessment of diastolic function, particularly in the presence of regional myocardial dysfunction [3, 4].

A similar algorithm could be employed using cardiac magnetic resonance imaging (MRI) volumetric function and 2D phase contrast imaging. MRI-based grading of diastolic dysfunction, however, lacks standardised acquisition protocols and evaluation methods, as well as clearly defined grading cutoffs, which are typically not interchangeable between methods [5–7]. Time-resolved three-dimensional three-directional phase contrast imaging (4D) flow MRI is increasingly recognised as a valuable modality for the noninvasive characterisation of cardiovascular hemodynamics and is becoming more widely used in routine cardiac MRI [8, 9]. Various studies demonstrated its superiority over standard 2D flow imaging in terms of accuracy for measuring blood flow velocities and volumes [10–13], particularly in the evaluation of valvular heart disease [14–16], cardiovascular shunts [17–19], parameters of diastolic function [12, 20], and the diagnosis and follow-up of complex congenital heart defects [9, 21, 22]. Additionally, 4D flow MRI has proven efficient in capturing and analysing blood flow at multiple locations, offering advantages in both acquisition and processing times [23]. With its ability to capture three-dimensional magnitude images along with the entire time-resolved, three-dimensional velocity field of the heart and surrounding vessels, 4D flow holds the potential for evaluating diastolic function from a single measurement. Furthermore, the a-posteriori nature of 4D flow data evaluation supports the possibility for standardisation and automation of diastolic function evaluation.

The purpose of this study was to investigate whether LV diastolic function could be evaluated from a single 4D flow measurement by employing the 2016 ASE/EACVI echocardiographic algorithm and to assess the accuracy of 4D flow-based grading of LV diastolic dysfunction using echocardiography as the reference standard.

## Materials and methods

### Study population

**Fig. 1:**
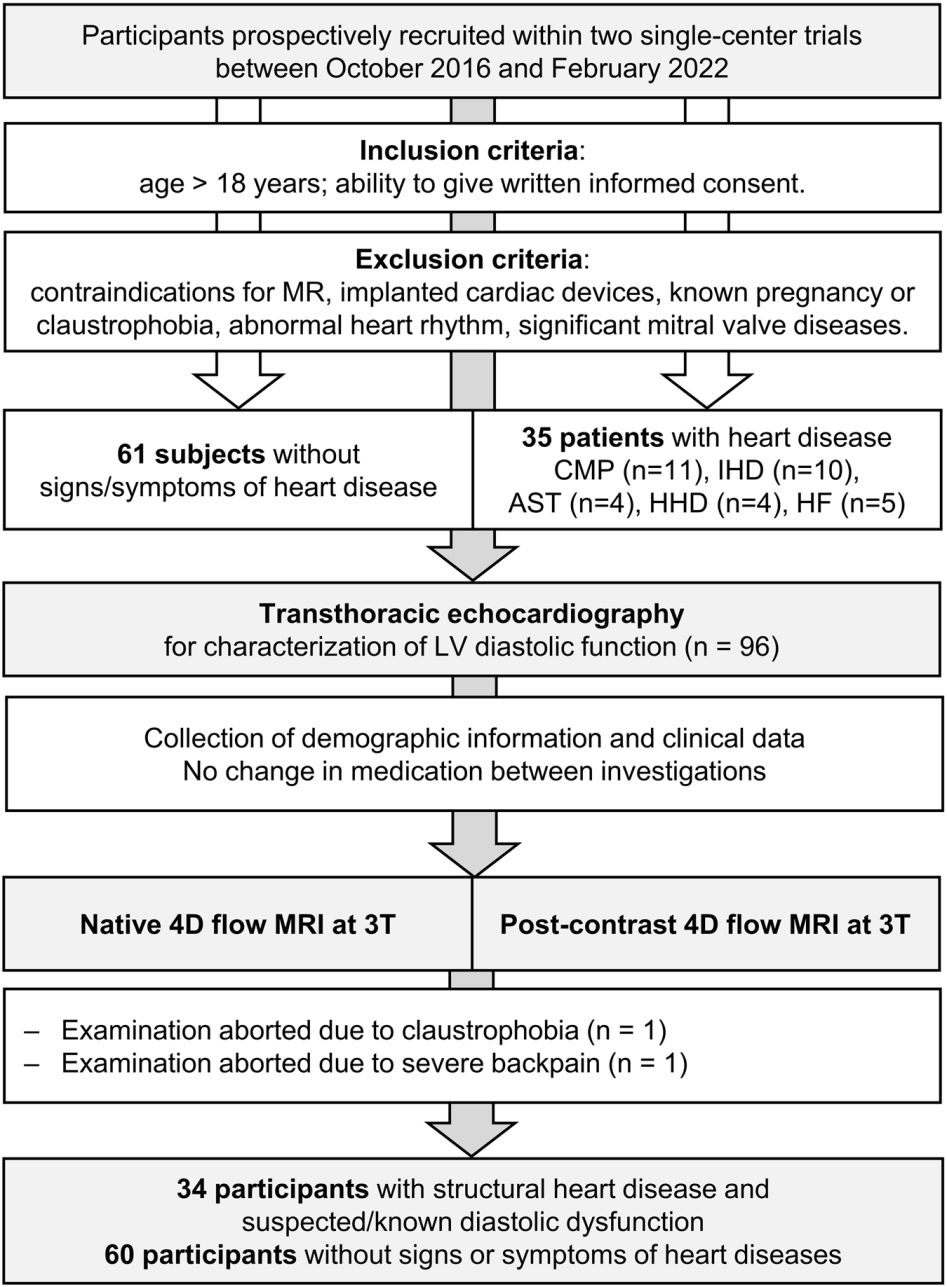
Study flow chart illustrating the participant selection process, inclusion and exclusion criteria, reasons for drop-out, data collection and size of the final study population. CMP, cardiomyopathy; IHD, ischaemic heart disease; AST, severe aortic stenosis; HHD, hypertensive heart disease; HF, heart failure

The study was approved by the local ethics review board and complied with the Declaration of Helsinki. Written informed consent was obtained from all participants. This study population was already investigated with regard to the role of the 4D flow-derived left atrial acceleration index [24], which can be considered a non-invasive marker for the presence of elevated LV filling pressure [25]. In short, 61 adult Caucasian participants without signs or symptoms of cardiac disease (ClinicalTrials.gov, NCT01728597) and 35 adult Caucasian participants with structural heart disease and suspected or known diastolic dysfunction (ClinicalTrials.gov, NCT03253835) were consecutively recruited between October 2016 and February 2022. Subjects with irregular heart rhythm or significant mitral valve stenosis were not enroled. All subjects were investigated by echocardiography for evaluation of LV diastolic (dys)function, as well as native (*n* = 61) or contrast-enhanced (*n* = 35) cardiac MRI, including 4D flow as part of a comprehensive imaging protocol. Two participants did not complete the MRI; thus, 94 participants were included in the analysis. Figure 1 shows the study flow chart and inclusion and exclusion criteria.

### Echocardiography

Transthoracic echocardiography, including Doppler and tissue Doppler imaging, was performed and evaluated according to the 2016 ASE/EACVI recommendations [2] by an experienced operator (C.R., 9 years of experience) using a Vivid E9 system (GE HealthCare) as previously described [20, 24]: The LV ejection fraction (EF) and maximal left atrial volume indexed to the body surface area (LAVI) were evaluated from apical 4-chamber and 2-chamber view series using the biplane Simpson method and the biplanar area-length method, respectively. Transmitral early (E) and late (A) diastolic peak velocities were measured in the apical 4-chamber view, and septal and lateral mitral annular peak velocities (e’) were acquired in the apical 4-chamber view. The evaluation of the tricuspid regurgitant peak velocity (TR) was performed with continuous-wave Doppler in the apical 4-chamber view. Parameter ratios, transmitral early-to-late diastolic peak velocity ratio (E/A) and E/e’ (using an average of septal and lateral e’) were calculated.

### 4D flow

All participants underwent 4D flow MRI under shallow breathing at 3 T (Magnetom Skyra, Siemens Healthineers). 4D flow data were acquired in 3-chamber-view orientation, using a 2D, retrospectively electrocardiographically or pulse-gated phase contrast sequence with three-directional velocity encoding [24], covering at least the LV, the left atrium and the main pulmonary artery. Protocol parameters were as follows: voxel size, 1.8 × 2.5 × 4.0 mm^3^; temporal resolution, 41.8 ms interpolated to 30 frames; echo time, 3.1 ms; flip angle, 12–15°; velocity encoding, 100–190 cm/s in all directions (adjusted to prevent aliasing); number of averages, 2. The typical acquisition time was 45 s per slice (50 heartbeats) or 22 min to cover the volume by 20–40 gapless slices.

4D flow data were evaluated by an experienced reader (C.R., 9 years of experience), blinded to the results from echocardiography, using standard software for volumetric analysis (cvi42, Circle Cardiovascular Imaging) and prototype software for evaluation of hemodynamic parameters (4Dflow, Siemens Healthineers). Parameters equivalent to those used in echocardiography for assessing diastolic (dys)function were derived, along with a surrogate parameter for TR indicating elevated pulmonary arterial pressure [2].

**Fig. 2:**
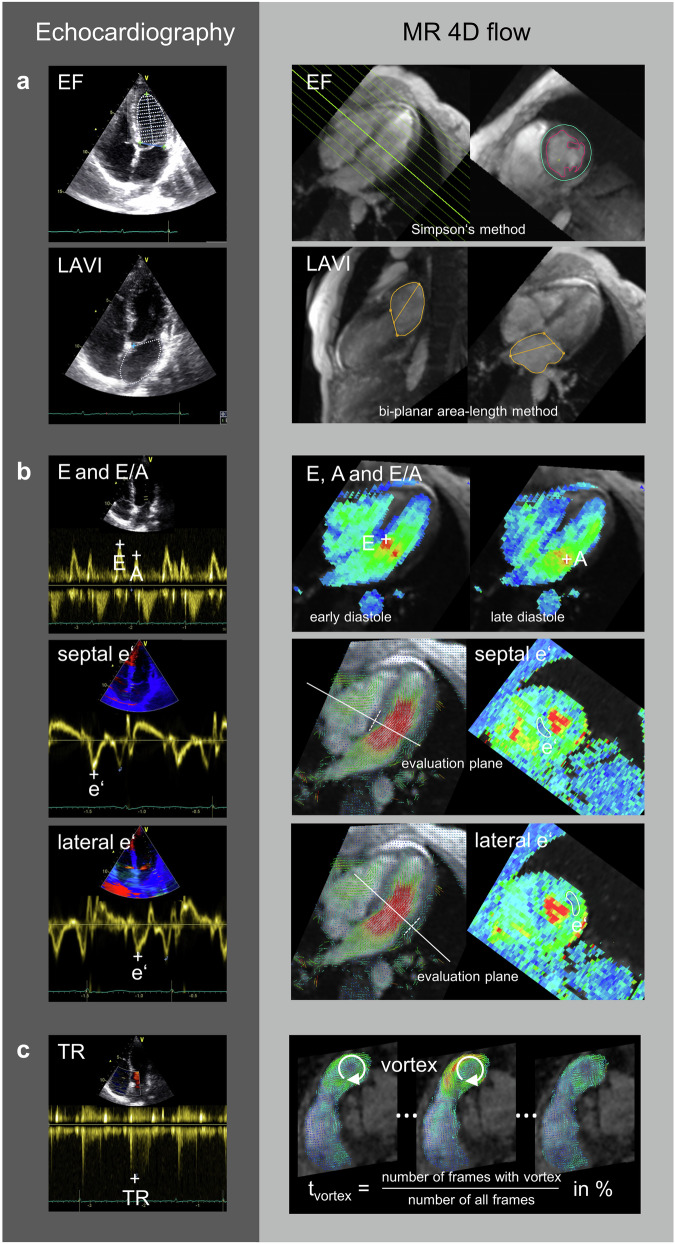
4D flow MRI-based assessment of parameters for the characterisation and grading of LV diastolic (dys)function according to the 2016 ASE/EACVI algorithm (right panel) with echocardiography as reference (left panel). **a** LV ejection fraction (EF) and left atrial volume index (LAVI) were evaluated from multiplanar reconstructed cut planes of 4D flow magnitude data. **b** Transmitral early (E) and late (A) diastolic peak velocities were assessed from the voxel with the highest velocity in the region between the mitral valve ring and the valve tips (white cross). Septal and lateral early diastolic myocardial peak velocities were evaluated as substitutes for echocardiographic early-diastolic mitral annular peak velocities in the basal LV myocardium as the peak average through-plane velocities in the posterior septal and the anterior lateral myocardium (white lines), respectively. **c** Duration of vortical blood flow along the main pulmonary artery (*t*_vortex_), indicating elevated pulmonary arterial pressure, was used as a surrogate parameter for echocardiographic TR. Arrows indicate the vortex in frames from the beginning, middle, and end of its existence

LV volumetric function, including the LV ejection fraction (EF), was evaluated by manual segmentation of the LV endo- and epicardial borders in a stack of multiplanar reconstructed cut planes in short-axis orientation using 4D flow magnitude data, as previously described [26]. Papillary muscles and trabeculae were excluded from the LV cavity. The LAVI was derived from manual segmentation of the left atrium in multiplanar reconstructed 4-chamber and LV 2-chamber view series using the biplanar area-length method (Fig. 2a).

E and A velocities were evaluated from the phase offset-corrected 4D velocity field based on the voxels with the highest velocity in the transmitral inflow region at early and late diastole [20]. Early diastolic septal, lateral and average e’ were determined as regionally averaged myocardial peak velocities through cut planes aligned perpendicular to the basal posterior septal or the basal anterior lateral myocardium, respectively (Fig. 2b).

Vortical blood flow along the main pulmonary artery, an indicator of elevated pulmonary arterial pressure, was evaluated visually using multiplanar reconstructed 3D velocity vector fields in right ventricular outflow tract orientation (Fig. 2c and Electronic Supplementary Material S1). The duration of vortical blood flow (*t*_vortex_) was defined as the percentage of cardiac frames with vortical blood flow in the cardiac interval, as previously described [27].

### Grading of diastolic (dys) function

Diastolic dysfunction was diagnosed and graded according to the 2016 ASE/EACVI algorithm using corresponding echocardiographic and 4D flow grading parameters. Grading cutoffs for 4D flow-derived metrics were chosen based on (1) adjustment for any possible bias between echocardiographic and 4D flow parameters in the case of echocardiography-equivalent grading parameters such as EF, LAVI, E, E/A, septal e’, lateral e’, and E/e’, and (2) a relationship derived between the peak tricuspid pressure gradient (pTR) = 4 ·TR^2^ (with pTR in mmHg and TR in m/s) and *t*_vortex_, introduced as a 4D flow surrogate parameter for TR.

### Interobserver variability

To investigate interobserver variability, MR 4D flow analysis was repeated by a second reader for the first five consecutive recruited subjects of each grade of diastolic dysfunction according to echocardiographic grading (resulting in 25 subjects), blinded to the results of the first read. Evaluation time, including pre- and post-processing of volumetric data, velocities, and pulmonary artery blood flow patterns, was measured from these evaluations.

### Statistical analysis

Statistical analysis was performed using SPSS^®^ (Version 27, IBM Corp) and NCSS (version 11, NCSS, LLC). Distributions of parameters are specified as means and standard deviations, categorical variables are reported as frequencies together with percentages, and diagnostic measures are specified together with 95% confidence intervals (CIs) in brackets. A *p*-value < 0.05 was regarded as statistically significant.

Normality of distributions was tested using the Shapiro–Wilk test. Continuous parameters of different groups of diastolic (dys)function were compared by analysis of variance with Dunett-T3 as post-hoc test or by Kruskal-Wallis test, as appropriate; percentages were compared by Pearson’s Chi-Square test. Relationships between corresponding parameters evaluated by echocardiography and 4D flow were assessed by Pearson’s correlation, Bland–Altman and accuracy analyses. Depending on the normality of differences, the significance of a bias was tested either with a one-sample *t*-test or with the Wilcoxon signed-rank test. The relationship between pTR and *t*_vortex_ was fitted to a segmented linear model, where *t*_vortex_ is 0% up to a threshold pTR_0_ of pTR and increases linearly from 0% for pTR ≥ pTR_0_.

Interobserver variability of parameters used for grading was quantified by a two-way mixed-effects model, single measure, absolute agreement intra-class correlation coefficients (ICC). ICCs between 0.75 and 0.9 are termed good, and ICC above 0.9 are termed excellent [28]. Agreement of grading of diastolic dysfunction between echocardiography and 4D flow, as well as interobserver variability, were evaluated using 5 × 5 contingency table analysis, with Cohen’s weighted kappa measuring agreement and the McNemar test being used to assess for evidence of a trend of grade overestimation or underestimation by either grading modality or reader.

Correlations were interpreted as high when the correlation coefficient was between 0.70 and 0.90, and very high if the correlation coefficient exceeded 0.90 [29]. A kappa above 0.80 was interpreted as almost perfect agreement [30].

## Results

### Study population

The characteristics and clinical data of the study population are summarised in Table 1. Data from all 94 participants were included in the analysis (Fig. 1). The mean age of the participants was 62 ± 12 years, with 50 participants (53%) being female. Echocardiography classified the diastolic function of 51 participants (54%) as normal. Diastolic dysfunction of grade I was diagnosed in 13 participants (14%), grade II in 13 participants (14%), and grade III in 8 participants (9%). In nine participants (10%), diastolic function was indeterminate. Echocardiography and 4D flow MRI were performed within 3 ± 7 days of each other. The mean heart rates of participants during echocardiography and 4D flow MRI did not differ significantly (*p* = 0.33).

**Table 1:** Participants’ demographic characteristics and left heart function parameters

Characteristics		All	Grade 0	Indet	Grade 1	Grade 2	Grade 3	*p*
		(*n* = 94)	(*n* = 51)	(*n* = 9)	(*n* = 13)	(*n* = 13)	(*n* = 8)	
Demography								
Age (years)		62 ± 12	61 ± 9	59 ± 8	58 ± 17	68 ± 15	65 ± 14	0.212
Sex female		50 (52)	29 (57)	7 (78)	5 (39)	6 (46)	3 (38)	0.326
Height (cm)		170 ± 10	171 ± 9	166 ± 8	172 ± 11	167 ± 10	171 ± 10	0.249
Weight (kg)		77 ± 15	75 ± 13	76 ± 21	80 ± 19	77 ± 13	86 ± 10	0.106
BMI (kg/m^2^)		26 ± 4	25 ± 3	28 ± 6	27 ± 4	28 ± 5	30 ± 4	0.082
BSA (m^2^)		1.90 ± 0.21	1.88 ± 0.20	1.86 ± 0.27	1.95 ± 0.28	1.88 ± 0.18	2.01 ± 0.15	0.237
sBP (mmHg)		136 ± 19	134 ± 17	144 ± 15	138 ± 20	140 ± 26	136 ± 20	0.578
dBP (mmHg)		76 ± 11	75 ± 10	77 ± 7	73 ± 9	77 ± 15	86 ± 12	0.790
Laboratory parameters								
Hb (g/L)		14 ± 2	14 ± 1	14 ± 1	15 ± 2	14 ± 1	13 ± 3	0.065
eGFR (mL/min)		76 ± 18	80 ± 10^3^	82 ± 13^3^	78 ± 25^3^	74 ± 22	48 ± 16^indet,1,2^	< 0.001
NTproBNP (pg/mL)		1298 ± 5986	76 ± 124^2,3^	84 ± 53^2,3^	561 ± 647^3^	689 ± 770^0,indet,3^	15,233 ± 18,651^0,indet,1,2^	< 0.001
Triglycerides (mg/dL)		113 ± 60	105 ± 49	97 ± 63	141 ± 49	132 ± 96	114 ± 76	0.075
HDL (mg/dL)		62 ± 20	65 ± 21	72 ± 23	50 ± 16	57 ± 12	48 ± 13	0.029
LDL (mg/dL)		135 ± 43	154 ± 31^1,2^	136 ± 45	99 ± 34^0^	92 ± 40^0^	121 ± 44	< 0.001
Functional status								
NYHA classification	I	63 (67)	51(100)	0 (0)	2 (15)	1 (8)	0 (0)	
	II	20 (21)	0 (0)	9 (100)	9 (70)	9 (69)	2 (25)	
	III	10 (11)	0 (0)	0 (0)	2 (15)	3 (23)	5 (63)	
	IV	1 (1)	0 (0)	0 (0)	0 (0)	0 (0)	1 (12)	
Depressed EF_Echo_		10 (11)	0 (0)	0 (0)	2 (15)	5 (38)	3 (38)	
MR 4D flow LV function								
Heart rate (s^−1^)		68 ± 11	69 ± 10	66 ± 8	69 ± 10	61 ± 11	74 ± 16	0.170
EF (%)		58 ± 11	62 ± 5^3^	61 ± 8	53 ± 15	55 ± 8	41 ± 16^0^	< 0.001
EDVI (mL/m^2^)		93 ± 18	88 ± 14	87 ± 10	97 ± 18	101 ± 21	110 ± 26	0.005
ESVI (mL/m^2^)		40 ± 17	33 ± 8	34 ± 10	47 ± 21	47 ± 18	65 ± 24	< 0.001
SVI (mL/m^2^)		52 ± 11	54 ± 8	46 ± 18	50 ± 12	55 ± 8	45 ± 17	0.059
CI (I/min/m^2^)		3.6 ± 1	3.7 ± 0.8	3.5 ± 0.5	3.5 ± 1.2	3.3 ± 0.6	3.3 ± 1.3	0.400
LVMI (g/m^2^)		79 ± 23	66 ± 12^1,2,3^	69 ± 9^1,2,3^	87 ± 10^0,indet,3^	99 ± 19^0,indet^	125 ± 20^0,indet,1^	< 0.001

Categorical data are presented as frequencies with percentages in parentheses; continuous data are presented as means with standard deviations in parentheses. *p*-value refers to the analysis of variance. Depressed EF_Echo_ refers to an ejection fraction < 50% assessed by echocardiography. Superscripts 0, indet, 1, 2, 3 indicate significant differences from grade 0, indeterminate, 1, 2, 3, respectively

*BMI* body mass index (BMI = weight/height^2^ with weight in kilograms and height in metres), *BSA* body surface area (BSA = weight^0.425^  × height^0.725^ × 0.007184 with weight in kilograms and height in metres), *sBP* systolic blood pressure, *dBP* diastolic blood pressure, *Hb* haemoglobin, *eGFR* estimated glomerular filtration rate, *NTproBNP* N-terminal prohormone of brain natriuretic peptide, *HDL* high-density lipoprotein, *LDL* low-density lipoprotein, *NYHA* New York Heart Association, *EF* ejection fraction, *EDVI* end diastolic volume indexed to BSA, *ESVI* end-systolic volume indexed to BSA, *SVI* stroke volume indexed to BSA, *CI* cardiac index, *LVMI*, left ventricular mass indexed to BSA

### Volumetric grading parameters

**Fig. 3:**
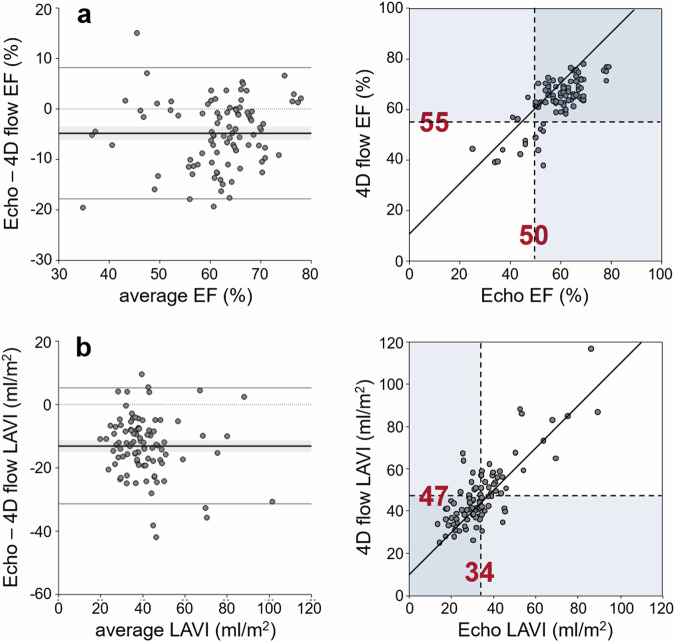
Bland–Altman plots and scatter plots comparing (**a**) ejection fraction and (**b**) left atrial volume index determined by echocardiography and 4D flow imaging. In the Bland–Altman plots, the dotted line indicates the line of identity, the bold solid line with the grey bars the bias with its 95% confidence interval, and the upper and lower solid lines the 95% limits of agreement. In the scatter plot, the bold line indicates the bias-adjusted line of identity. Echocardiographic grading cutoff and bias-adjusted 4D flow grading cutoff are indicated by dashed lines. Echo, echocardiography; EF, ejection fraction; LAVI, left atrial volume index

While high correlations between volumetric parameters assessed by both modalities were observed, 4D flow-derived values exceeded echocardiographic values significantly (Table 2 and Fig. 3). Bias-adjustment of the established echocardiographic grading cutoffs of 50% for LV ejection fraction (EF) and 34 mL/m^2^ for LAVI [2] led to grading cutoffs of 55% and 47 mL/m^2^ for 4D flow-derived EF and LAVI, respectively. Accuracies for predicting echocardiographic EF < 50% and LAVI > 34 mL/m^2^ by corresponding 4D flow parameters and their bias-adjusted grading cutoffs were 91% (83%, 96%) for EF and 77% (67%, 85%) for LAVI.

**Table 2:** Comparison of volumetric grading parameters derived from echocardiography and 4D flow

Parameter	Echo	4D flow	Bias	*p*	r
EF (%)	59 ± 10	64 ± 9	−5	< 0.001	0.75
LAVI (mL/m^2^)	35 ± 14	48 ± 15	−13	< 0.001	0.80

Parameters are presented as means ± standard deviations. The *p*-value refers to the significance of the bias; *r* to the correlation between echocardiographic and 4D flow measurements

*EF* ejection fraction, *LAVI* left atrial volume index, *Echo* echocardiography

### Diastolic transmitral flow and myocardial velocity grading parameters

**Fig. 4:**
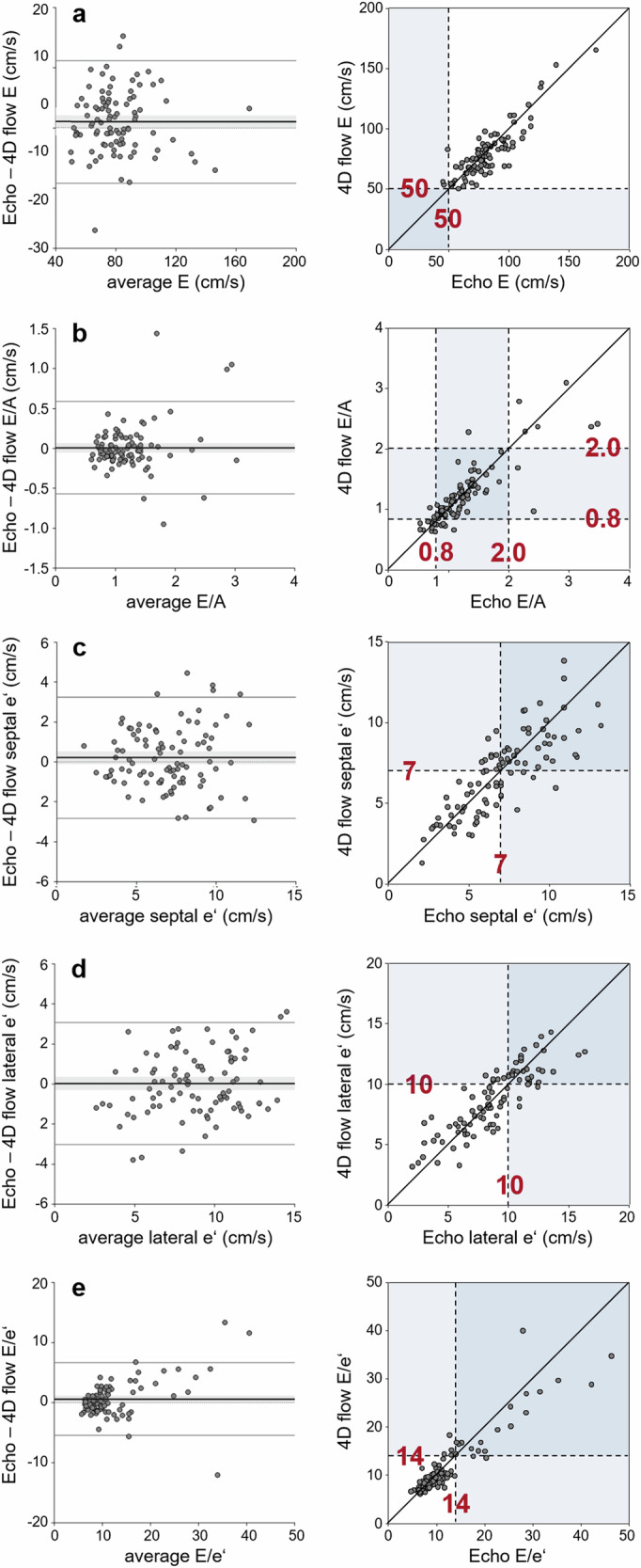
Bland–Altman plots and scatter plots comparing (**a**) early diastolic transmitral peak velocities, (**b**) the ratio of early-to-late diastolic transmitral peak velocities, (**c**) septal early diastolic mitral annular/myocardial peak velocity, (**d**) lateral early diastolic mitral annular/myocardial peak velocity, and **e** the ratio of early diastolic transmitral peak velocity to average early diastolic mitral annular/myocardial peak velocity determined by echocardiography and MRI. In the Bland–Altman plots, the dashed line indicates the line of identity, the bold solid line with the grey bars the bias with its 95% confidence interval, and the upper and lower solid lines the 95% limits of agreement. In the scatter plot, the bold line indicates the (bias-adjusted) line of identity. Echocardiographic grading cutoffs and 4D flow grading cutoffs are indicated by dotted lines. Echo, echocardiography; E, early diastolic transmitral peak velocity; E/A, ratio of early-to-late diastolic transmitral peak velocities; e’, early diastolic mitral annular/myocardial peak velocity; E/e’, ratio of early diastolic transmitral peak velocity to average early diastolic mitral annular/myocardial peak velocity

Correlations between E, E/A, septal and lateral e’, as well as E/e’ values obtained from echocardiography and from 4D flow ranged from high to very high and demonstrated no significant bias (Table 3 and Fig. 4). As a consequence, bias-adjusted 4D flow grading cutoffs were chosen that were identical to the established echocardiographic thresholds: 50 cm/s for E, 0.8 and 2.0 for E/A, 7 cm/s for septal e’, 10 cm/s for lateral e’, and 14 for E/e’ [2]. Accuracies for predicting echocardiographic E > 50 cm/s, E/A > 0.8, E/A < 2.0, septal e’ < 7 cm/s, lateral e’ < 10 cm/s, and E/e’ > 14 by analogous 4D flow-based criteria were 97% (91%, 99%), 91% (83%, 96%), 97% (91%, 99%), 78% (68%, 86%), 88% (80%, 94%), and 93% (85%, 97%), respectively.

**Table 3:** Comparison of velocity parameters derived from 4D flow MRI and echocardiography

Parameter	Echo	4D flow	Bias	*p*	*r*
E (cm/s)	82 ± 21	80 ± 21	2.00	0.065	0.87
E/A	1.23 ± 0.54	1.23 ± 0.48	0.01	0.820	0.84
Septal e‘ (cm/s)	7.0 ± 2.5	6.8 ± 2.4	0.20	0.201	0.80
Lateral e‘ (cm/s)	8.8 ± 3.0	8.8 ± 2.7	0.00	0.884	0.86
E/e‘	12.3 ± 7.7	11.7 ± 6.4	0.60	0.069	0.92

Parameters are presented as means ± standard deviations

*E* early diastolic transmitral peak velocity, *E/A* early-to-late diastolic transmitral peak velocity ratio, *e’* early diastolic mitral annular/myocardial peak velocity, *Echo* echocardiography, *p* refers to the significance of the bias, *r* to the correlation between echocardiographic and 4D flow measurements

### Elevated pulmonary artery pressure grading parameter

**Fig. 5:**
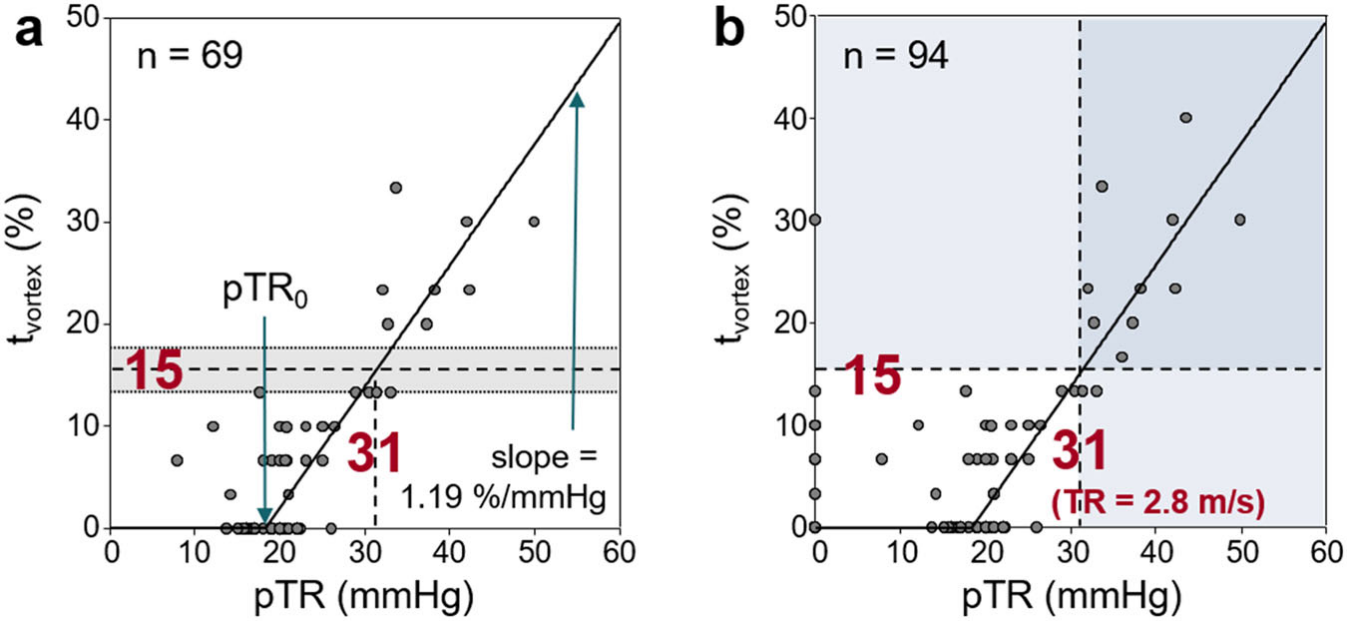
Scatter plot of 4D flow-derived duration of vortical blood flow along the main pulmonary artery vs the echocardiographic pTR in (**a**) participants with detectable tricuspid regurgitation and (**b**) all participants. The solid line demonstrates the fitted segmented linear model for the dependency of *t*_vortex_ on pTR with the fitting parameters pTR_0_ and slope. The vertical dashed lines indicate the echocardiographic grading cutoff for TR, the horizontal dashed lines the derived 4D flow grading cutoff for *t*_vortex_ in **a**, together with the 95% confidence interval of the model-based estimate, shown as a grey bar and dotted lines. In **b**, pTR was interpreted as 0 if there was no detectable tricuspid regurgitation. pTR, peak tricuspid pressure gradient; *t*_vortex_, vortex duration along the main pulmonary artery; TR, tricuspid regurgitant peak velocity; pTR_0_, threshold value of the segmented linear model

Tricuspid regurgitation was found by echocardiography in 69 (73%) of the 94 subjects. In these 69 subjects, the relationship between echocardiographic pTR and 4D flow MRI-derived *t*_vortex_ as surrogate grading parameter was well described (square root of the coefficient of determination *R* = 0.88) by a segmented linear model with threshold pTR_0_ = 18.4 mmHg and slope = 1.19 %/mmHg (Fig. 5a). This model predicts *t*_vortex_ = 15% (13%, 17%) for the echocardiographic grading cutoff TR = 2.8 m/s or, equivalently, pTR = 31.4 mmHg, which was chosen as a grading cutoff for 4D flow measurements. The accuracy for predicting echocardiographic TR > 2.8 m/s by 4D flow-derived *t*_vortex_ > 15% for all subjects with tricuspid regurgitation detectable on echocardiography was 99% (92%, 100%). As there was one subject with *t*_vortex_ > 15% without visible tricuspid regurgitation (Fig. 5b), this accuracy was reduced to 98% (93%, 100%) for all subjects. Of note, only trace TR was detected on 4D flow MRI in this subject.

### Evaluation time

The time required to evaluate EF and LAVI from multiplanar reconstructed magnitude series was 8 ± 2 min. Assessing diastolic transmitral and myocardial tissue velocities (including pre-processing) took 10 ± 2 min; visual evaluation of *t*_vortex_ required 6 ± 1 min, resulting in a total evaluation time of less than 30 min.

### Grading of diastolic dysfunction

**Fig. 6:**
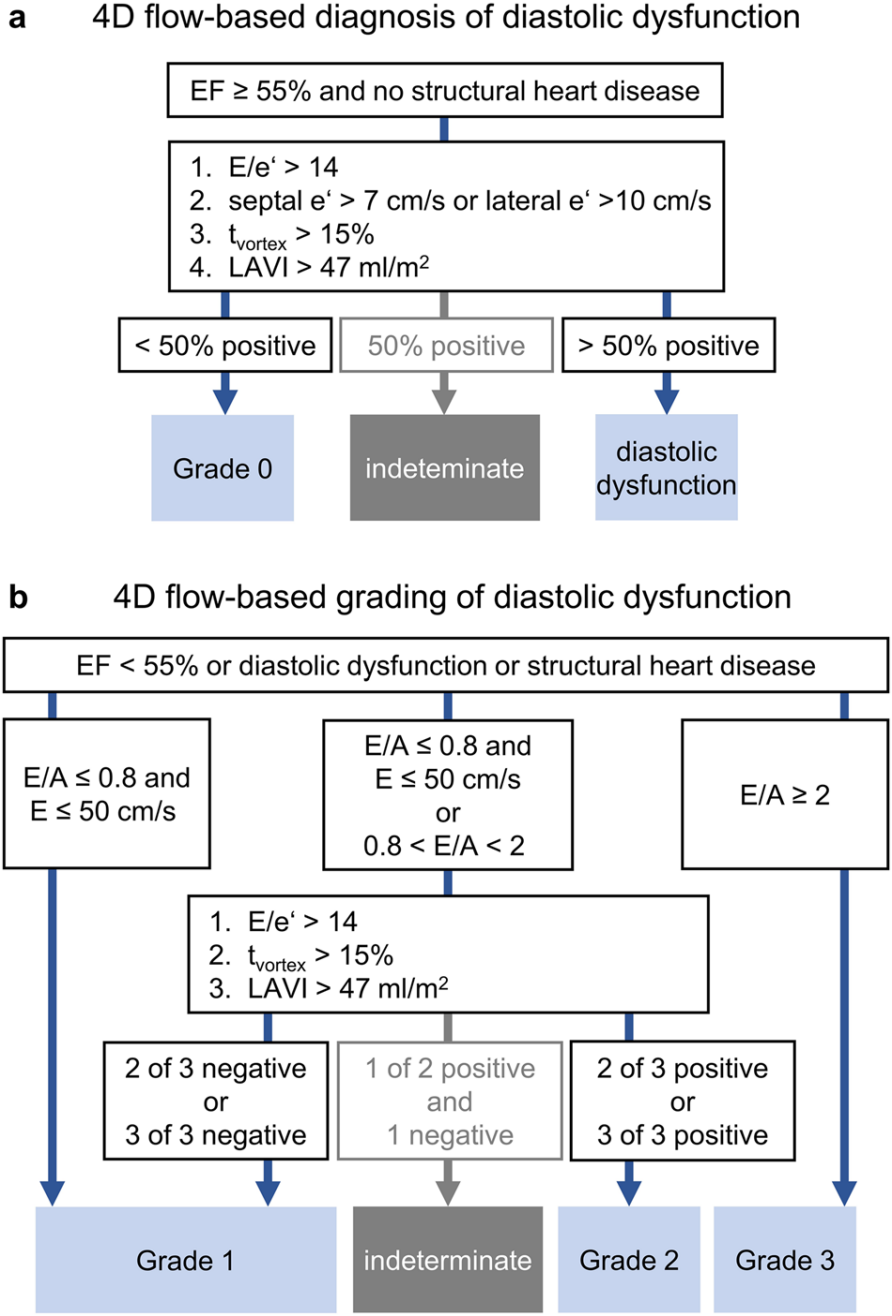
Algorithm for the echo-equivalent diagnosis (**a**) and grading (**b**) of LV diastolic dysfunction using 4D flow following the echocardiographic, multiparametric, threshold-based 2016 ASE/EACVI algorithm, along with the 4D flow parameter thresholds. EF, left ventricular ejection fraction; LAVI, maximal left atrial volume index; E, transmitral early diastolic peak velocity; E/A, early-to-late diastolic peak velocity ratio; e’, early diastolic myocardial peak velocity; average E/e’, ratio of transmitral early diastolic peak velocity and the average of septal and lateral early diastolic myocardial peak velocities; *t*_vortex_, duration of vortical blood flow along the main pulmonary artery

Figure 6 summarises the adapted 2016 ASE/EACVI algorithm for evaluation of diastolic dysfunction from 4D flow grading parameters and their cutoffs (introduced above). Most discrepancies between echocardiography and 4D flow MRI-based grading were caused by differences in the categorisation of LAVI, accounting for 13 of 21 cases. In five cases, different grading resulted from different categorisation of early diastolic mitral annular/myocardial tissue velocity. Notably, all of these were borderline cases, either meeting or missing the cutoff by less than 1 cm/s. In three cases, discrepancies were due to E/A ratio differences between modalities. However, only one case exhibited significant disagreement with E/A = 2.2 by echocardiography vs 1.3 by 4D flow MRI. The comparison of echocardiographic and 4D flow-based grading of diastolic dysfunction (Table 4) demonstrated almost perfect agreement with a weighted kappa of 0.84 (0.78, 0.91). There was no evidence for over- or underestimation of grades by the 4D flow approach (*p* = 0.53).

**Table 4:** Comparison of echocardiographic and 4D flow MRI-based grading of LV diastolic dysfunction

		Echocardiography					
	Grade	0	Indet	1	2	3	Total
4D flow	0	44	6	0	0	0	50
	Indet	7	3	0	0	0	10
	1	0	0	12	4	0	16
	2	0	0	1	8	2	11
	3	0	0	0	1	6	7
	Total	51	9	13	13	8	94

*Grade 0* normal LV diastolic function, *indet* indeterminate

### Interobserver variability

The 4D flow parameters used for grading displayed excellent interobserver agreement, except septal e’, which displayed only good agreement (Table 5). Agreement of grading between readers was almost perfect with a weighted kappa of 0.97 (0.92, 1.00). There was only disagreement for one subject between normal and indeterminate diastolic dysfunction (Electronic Supplementary Material S2). Of note, none of the subjects who were classified as diastolic dysfunction grade 2 by echocardiography and grade 1 by the first reader were reclassified in the repeated evaluation.

**Table 5:** Interobserver variability for 4D flow grading parameters of LV diastolic dysfunction assessed from a subset of 25 participants

Parameter	ICC
EF	0.961 [0.799–0.987]
LAVI	0.992 [0.982–0.996]
E	0.986 [0.967–0.994]
E/A	0.984 [0.964–0.993]
e‘ septal	0.861 [0.711–0.936]
e‘ lateral	0.925 [0.838–0.966]
E/e‘	0.958 [0.906–0.981]
*t*_vortex_	0.976 [0.946–0.989]

Intraclass correlation coefficients (ICCs) are given with corresponding 95% confidence intervals in brackets

*EF* ejection fraction, *LAVI* left atrial volume index, *E* early diastolic transmitral peak velocity, *E/A* early-to-late diastolic peak velocity ratio, *e’* early diastolic septal and lateral myocardial peak velocities, *t*_vortex_ duration of vortical blood flow along the main pulmonary artery

Furthermore, in the same subset of patients, grading of diastolic dysfunction was investigated by LAVI and EF derived from cine-bSSFP images instead of 4D flow magnitude images, leading to similar but not identical grading of diastolic dysfunction (Electronic Supplementary Material S3).

## Discussion

Our study demonstrated that grading of LV diastolic dysfunction from 4D flow MRI, using an approach modelled on the 2016 ASE/EACVI algorithm for evaluation of diastolic dysfunction by echocardiography, achieved nearly perfect agreement with the latter method. The 4D flow approach involved (1) selection of bias-adjusted 4D flow grading thresholds for volumetric parameters, (2) selection of echo-equivalent grading cutoffs for diastolic transmitral and myocardial peak velocities, as well as velocity ratios, and (3) use of the duration of vortical blood flow along the main pulmonary artery *t*_vortex_ > 15% as a substitute grading cutoff for the echocardiographic TR > 2.8 m/s.

Cardiac MR represents the established reference method for assessment of ventricular and atrial volumetric function from cine imaging [31]. EF and LAVI derived from multiplanar-reconstructed 4D flow magnitude data have shown high correlations and comparable inter- and intraobserver variability with standard cine balanced steady-state free precession-derived volumetric function parameters [26]. As expected, and in accordance with previous studies comparing echocardiographic EF with EF derived from cardiac MRI [32–34], we observed a high correlation between EF derived from 4D flow and from echocardiography. 4D flow-derived EF was evaluated by manual segmentation of the LV endocardial borders, with papillary muscles and trabeculae excluded from the LV cavity [31]. This might explain the small but significant bias, which was taken into account in the bias-adjusted cutoff for EF. Consistent with literature comparing LAVI determined from echocardiography and cardiac MRI, we observed a high correlation between values from the two modalities but significant underestimation of LAVI by echocardiography [35, 36]. This underestimation has previously been attributed to poor left atrial endocardial wall definition and foreshortening in echocardiography [35].

Whereas biases for volumetric grading parameters had to be considered, 4D flow allowed assessment of diastolic transmitral flow and myocardial velocity grading parameters without any significant bias to echocardiography. High correlations between echocardiographic and 2D, as well as 4D flow-derived E, E/A, e’, and E/e’ values have been reported in different studies [12, 20, 37–41], in which 2D measurements tended to underestimate echocardiographic E, A and e’ [20, 38–41]. 4D flow MRI, allowing detection of the highest transmitral velocities in the whole inflow volume on the one hand and a posteriori optimisation of myocardial cutplanes on the other hand, made it possible to circumvent the problem of underestimation of echocardiographic E, A and e’ [12, 20], explaining the insignificant differences between echocardiographic and 4D flow-derived E, E/A, e’ and E/e’ values in the current study.

Although phase contrast imaging could be used to measure TR equivalent to that from echocardiography [42], it would necessitate the acquisition of an additional high VENC 4D flow measurement; therefore, the current study employed a surrogate grading parameter for TR, as suggested previously [43]. TR—or more precisely the pTR—equals, up to the right atrial pressure, the systolic pulmonary arterial pressure [44], and the cutoff TR > 2.8 m/s is also commonly used to suggest the presence of pulmonary hypertension [45]. The 4D flow-derived duration of vortical blood flow along the main pulmonary artery has been shown to demonstrate a segmented linear relationship to the mean pulmonary arterial pressure, where elevated mean pressure (in mmHg) can be estimated accurately from *t*_vortex_ via 16 + 0.63·*t*_vortex_ [27]. Given the fact that systolic and mean pulmonary arterial pressures are approximately proportional [46], the close relationship between pTR and *t*_vortex_ found in the current study is understandable (see also Electronic Supplementary Material S4). The model-derived 4D flow grading cutoff *t*_vortex_ > 15% used as a surrogate for the echocardiographic TR > 2.8 m/s corresponds to a mean pulmonary arterial pressure cutoff of 25 mmHg, which is the (former) threshold for the presence of pulmonary hypertension [45]. Using the above grading cutoffs, the agreement between 4D flow and echocardiography in predicting elevated pulmonary arterial pressures was high, both in participants with detectable tricuspid regurgitation, as well as in the entire study population (when interpreting non-detectable regurgitation as TR ≤ 2.8 m/s). Notably, the given grading cutoff *t*_vortex_ > 15% (recalculated to a mean pulmonary arterial pressure > 25 mmHg) is not necessarily the optimal cutoff for 4D flow-based detection of pulmonary hypertension. A reduction of the threshold to *t*_vortex_ > 13% (recalculated to a mean pulmonary arterial pressure > 24 mmHg) would still lie within the CI of the model estimate of TR = 2.8 m/s and would even better match the definition of pulmonary hypertension. The lower cutoff would, however, reduce the agreement between 4D flow and echocardiography, which is in accordance with the observation that *t*_vortex_ more accurately depicts elevated mean pulmonary arterial pressure than does echocardiography when compared to right heart catheter measurements [47].

In contrast to echocardiography, 4D flow MRI provides an operator-independent method for assessment of comprehensive flow dynamics, offering reproducible assessment of diastolic function, particularly in cases where echocardiographic results are inconclusive or technically challenging [3, 4]. Unlike the echocardiographic 2016 ASE/EACVI algorithm, the proposed echo-equivalent 4D flow algorithm does not require a separate distinction for cases where not all grading parameters are assessed [2]. Since all grading parameters are derived from a single measurement and evaluated a posteriori, individual parameters are not susceptible to omission, as may occur with echocardiographic or comprehensive cardiac MR protocols based on cine function, 2D, and 4D phase contrast imaging [41, 43]. Moreover, using advanced cardiac MRI myocardial function and tissue characterisation supplements echocardiography, allowing identification of specific cardiac pathologies [48]. While outside the scope of this study, it is tempting to speculate that incorporating tissue characterisation has the potential to supplement or simplify the algorithm. The observed agreement in grading between echocardiography and 4D flow is comparable to the agreement found with repeated echocardiographic evaluation described previously [49], which represents the upper bound of achievable agreement between methods.

Our study had certain limitations: Though prospective, it was a medium-sized single-centre study. Echocardiography and 4D flow data were not acquired simultaneously, and this could potentially have introduced differences between parameters. However, non-simultaneous evaluation of clinically relevant LV diastolic dysfunction is assumed to have been acceptable, as there were no changes in medication between investigations, particularly vasodilator therapy or diuretics, which could have influenced assessed parameters [49]. Patients with irregular heart rhythm were excluded from the study, because echocardiographic real-time measurement and segmented 4D flow data acquisition might have caused methodological biases in the derived grading metrics; this especially limits the applicability of results to patients with atrial fibrillation. Assessment of the interscan agreement of 4D flow MRI-based grading was beyond the scope of this study. In the present study, bias-adjusted 4D flow grading cutoffs were applied for volumetric, transmitral, and myocardial velocity parameters; the observed agreement in diastolic dysfunction grading may vary when other strategies for cutoff definition are used. Moreover, grading cutoffs for EF and LAVI may differ when evaluated from standard cine bSSFP series instead of 4D flow magnitude images. The present study was performed at 3 T MR; previous research on the impact of field strength on 4D flow MRI showed good agreement between peak velocities at 1.5 T and 3 T, similar enough to be interchangeable for comparable sequences [50, 51]. This study was intended as a proof-of-concept study. Manual evaluation of volumetric and hemodynamic 4D flow data was employed, resulting in evaluation times of approximately 30 min, together with long scan times, limiting broader clinical applicability. However, rapidly evolving 4D flow acceleration [9] and automated evaluation techniques [52–54] could aid clinical application in the future.

In conclusion, grading of LV diastolic dysfunction from a single 4D flow measurement is feasible and shows nearly perfect agreement with echocardiography. 4D flow MRI-based grading of LV diastolic dysfunction could not only serve as a viable noninvasive alternative to echocardiography in patients with poor echocardiographic image quality and those referred for cardiac MRI during a diagnostic workup, but could also facilitate validation of new cardiac MRI and 4D flow parameters of advanced myocardial tissue and hemodynamical characterisation for assessing LV diastolic dysfunction in future studies using concurrently acquired data.

## Supplementary information

ELECTRONIC SUPPLEMENTARY MATERIAL

## Abbreviations

4D flow: Time-resolved three-dimensional three-directional phase contrast imaging
ASE: American Society of Echocardiography
E: Transmitral early diastolic peak velocity
E/A: Transmitral early-to-late diastolic peak velocity ratio
e’: Early diastolic mitral annular/myocardial peak velocity
EACVI: European Association of Cardiovascular Imaging
EF: Left ventricular ejection fraction
LAVI: Maximal left atrial volume index
LV: left ventricle or left ventricular
MRI: Magnetic resonance imaging
pTR: Peak tricuspid pressure gradient
TR: Tricuspid regurgitant peak velocity
*t*_vortex_: 4D flow-derived vortex duration along the main pulmonary artery
