# Supplementary material for: 4D flow MRI-based grading of left ventricular diastolic dysfunction: a validation study against echocardiography
Source: Eur Radiol. 2025 May 25;35(11):7118–32. doi: 10.1007/s00330-025-11703-0 (PMC12559040; doi:10.1007/s00330-025-11703-0)
Supplement: Supplementary file 1 — ELECTRONIC SUPPLEMENTARY MATERIAL [file 330_2025_11703_MOESM1_ESM.pdf]

4D flow MRI-based grading of left ventricular diastolic dysfunction: A validation study against echocardiography

ELECTRONIC SUPPLEMENTARY MATERIAL

S1 Evaluation of vortical blood flow along the main pulmonary artery

The acquired velocity fields were calculated and pre-processed, including phase offset error correction and phase unwrapping, as well as semi-automated segmentation of the pulmonary artery using dedicated software (4DFlow, Siemens Healthineers).

Vortices along the main pulmonary artery were analyzed visually from multiplanar reformatted 3D velocity vector fields in RVOT orientation for the presence of a PH-associated vortex (**Fig. S1**): A vortex was classified as PH-associated if velocity vectors formed closed, concentric, ring-shaped tangent curves along the main pulmonary artery [1, 2]. Vortices near the pulmonary valves, helical flow into the pulmonary branches, and vortices not formed during systole were excluded [2, 3]. The duration of the PH-related vortical blood flow (t_{vortex}) was assessed as time from the cardiac phase of vortex onset to dissolution.

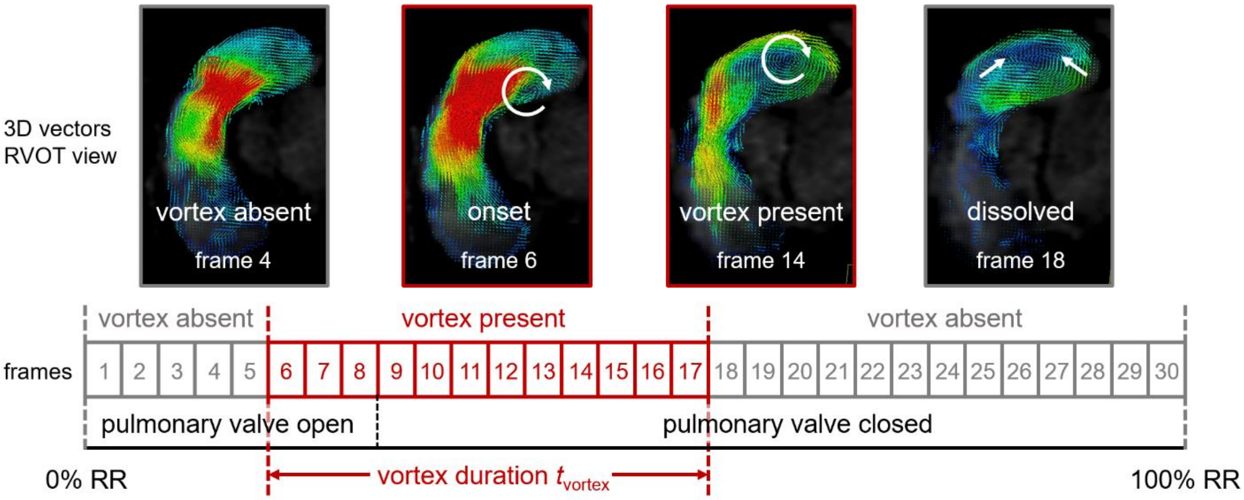

Figure S1: Visual assessment of duration of vortical blood flow along the main pulmonary artery. Vortical blood flow was analyzed using 3D velocity vectors multiplanar reconstructed in right ventricular outflow tract (RVOT) orientation. Time of vortex onset and time of vortex dissolution were the first and last cardiac frame showing PH-associated vortical blood flow (red frames), duration of vortical blood flow t_{vortex} was calculated relative to the number of frames of the cardiac interval (RR).

S2 Interobserver variability

Grading agreement is shown in the 5x5 contingency table, comparing the classifications assigned by two independent readers across all five grading categories or diastolic dysfunction (**Table S1**). The results demonstrate almost perfect agreement between the two observers. Discrepancies were minimal, with only a single disagreement involving the classification of one subject between normal and indeterminate diastolic dysfunction.

Table S1: Interobserver comparison of 4D flow MRI-based grading of left ventricular dysfunction.
Grade 0, normal LV diastolic function; indet, indeterminate.

	Reader 1						
	Grade	0	indet	1	2	3	total
Reader 2	0	**5**	0	0	0	0	5
	indet	1	**4**	0	0	0	5
	1	0	0	**9**	0	0	9
	2	0	0	0	**2**	0	2
	3	0	0	0	0	**4**	4
	total	6	4	9	2	4	25

S3 Cine bSSFP volumetric assessment of EF and LAVI

Volumetric parameters were derived from free breathing realtime steady state free precision (bSSFP) cine images acquired in both, long-axis and gapless short-axis series covering the left ventricle (LV). Typical imaging parameters were: spatial resolution $2.3 \times 3.9 \times 7$ mm^3 and $2.5 \times 4.2 \times 8$ mm^3 for long- and short-axis, respectively; echo time 1.5 ms; flip angle, 40°; parallel acquisition factor, 3; temporal resolution, 36 ms.

Image evaluation was performed using standard software (cvi42, Circle Cardiovascular imaging). Cine series were segmented semiautomatically, following the current guidelines [4]. The left ventricular ejection fraction (EF) was calculated from end-diastolic and end-systolic volumes; papillary muscles were excluded from the blood pool. The left atrial volume index (LAVI) was assessed by semiautomatically segmentation of the left atrial area and length at end-systole from both the LV 4-chamber and 4-chamber view. LAVI was calculated using the bi-planar area length method.

Grading of diastolic dysfunction was performed analogous to the 4D flow algorithm. Comparisons between 4D flow magnitude and cine bSSFP data were performed using paired t-test and correlation analysis. Agreement between grading of diastolic dysfunction using 4D flow magnitude and cine bSSFP-derived volumetric parameter was evaluated using 5x5 contingency table analysis with Cohen's weighted kappa measuring the degree of agreement.

LAVI values derived from cine bSSFP series and 4D flow magnitude images demonstrated a very high correlation between ($r = 0.91$, $p < 0.001$) without significant bias. Similarly, EF showed a high correlation ($r = 0.83$, $p < 0.001$); however, significant higher values were derived from 4D flow magnitude data (6.25 ± 7.5, $p < 0.001$). Grading agreement between 4D flow magnitude and cine bSSFP-derived volumetric parameter is shown in **Table S2**, demonstrating almost perfect agreement with a weighted kappa of 0.97 (0.91, 1.00).

Table S2: Comparison of grading of left ventricular diastolic dysfunction derived from 4D flow magnitude images and cine bSSFP series. Grade 0, normal LV diastolic function; indet, indeterminate.

		4D flow					
	Grade	0	indet	1	2	3	total
cine-bSSFP	0	**6**	0	0	0	0	6
	Indet	0	**4**	0	0	0	4
	1	0	0	**8**	0	0	8
	2	0	0	1	**2**	0	3
	3	0	0	0	0	**4**	4
	Total	6	4	9	2	4	25

The observed bias in EF has been previously reported and is likely due to differences in delineation of the endomyocardial contour at flash readout [5]. Notably, grading difference was observed in one subject, where higher LAVI measurements in cine bSSFP images resulted in a different classification. This finding suggests that while volumetric assessment between 4D flow magnitude images and cine bSSFP are closely aligned, the classification of LV diastolic dysfunction may differ depending on the imaging workflow used.

S4 Segmented linear model for prediction of the echocardiographic peak tricuspid pressure gradient from t_{vortex}

To establish the relationship between the duration of vortical blood flow along the main pulmonary artery t_{vortex} and the echocardiographic peak tricuspid pressure gradient pTR, a segmented linear regression model was employed. The model is based on empirical relationships between t_{vortex} and pTR.

Given the empirical formula describing the relationship between the mean pulmonary arterial pressure (mPAP) and t_{vortex} [2]:

$$\text{mPAP (mmHg)} \begin{cases} \leq 16 & \text{for } t_{vortex} = 0\% \\ = 16 + 0.63 \cdot t_{vortex} & \text{for } t_{vortex} > 0\% \end{cases}$$

the empirically given linear relationships between mPAP and the mean systolic pulmonary pressure sPAP = $^2/_3$·mPAP [6], and sPAP ≈ pTR [7], t_{vortex} relates directly to pTR by the segmented linear model:

$$t_{vortex} = \begin{cases} 0 & \text{for pTR} < \text{pTR}_0 \\ \alpha \cdot (\text{pTR}-\text{pTR}_0) & \text{for pTR} \geq \text{pTR}_0 \end{cases}$$

with t_{vortex} as percentage of the cardiac cycle, pTR as echocardiographic peak tricuspid pressure gradient (in mmHg) calculated from pTR = 4·TR^2, pTR_0 is the threshold value of pTR below which no vortical flow is observed, and α is the slope of the linear relationship between pTR and t_{vortex} beyond pTR_0.

Model parameters were assessed via a least-squares segmented regression analysis. The threshold was determined iteratively by minimizing the residual sum of squares. The derived threshold was pTR_0 = 18.4 mmHg and the slope α = 1.19% per mmHg, with a high coefficient of determination R^2 = 0.88. The model-derived threshold for t_{vortex} > 15% corresponds to the echocardiographic TR > 2.8 m/s (or pTR > 31.4 mmHg).

References

1. Reiter G, Reiter U, Kovacs G, et al (2008) Magnetic Resonance–Derived 3-Dimensional Blood Flow Patterns in the Main Pulmonary Artery as a Marker of Pulmonary Hypertension and a Measure of Elevated Mean Pulmonary Arterial Pressure. Circ: Cardiovascular Imaging. DOI:10.1161/CIRCIMAGING.108.780247
2. Reiter G, Reiter U, Kovacs G, et al (2015) Blood Flow Vortices along the Main Pulmonary Artery Measured with MR Imaging for Diagnosis of Pulmonary Hypertension. Radiology. DOI:10.1148/radiol.14140849
3. Reiter U, Reiter G, Fuchsjäger M (2016) MR phase-contrast imaging in pulmonary hypertension. BJR. DOI:10.1259/bjr.20150995
4. Schulz-Menger J, Bluemke DA, Bremerich J, et al (2020) Standardized image interpretation and post-processing in cardiovascular magnetic resonance - 2020 update. Journal of Cardiovascular Magnetic Resonance. DOI:10.1186/s12968-020-00610-6
5. Reiter C, Reiter G, Kräuter C, et al (2024) Evaluation of left ventricular and left atrial volumetric function from native MR multislice 4D flow magnitude data. Eur Radiol. DOI:10.1007/s00330-023-10017-3
6. Chemla D, Castelain V, Provencher S, et al (2009) Evaluation of Various Empirical Formulas for Estimating Mean Pulmonary Artery Pressure by Using Systolic Pulmonary Artery Pressure in Adults. Chest. DOI:10.1378/chest.08-0904
7. Chemla D, Castelain V, Herve P, et al (2002) Haemodynamic evaluation of pulmonary hypertension. European Respiratory Journal. DOI:10.1183/09031936.02.00068002
